# Determinants of serum concentration of first-line anti-tuberculosis drugs from China

**DOI:** 10.1097/MD.0000000000017523

**Published:** 2019-10-11

**Authors:** Qian Lei, Hao Wang, Yuan Zhao, Liyun Dang, Changsheng Zhu, Xiaohui Lv, Hui Wang, Jun Zhou

**Affiliations:** a Department of Pharmacy; b Department of Medical, Xi’an Chest Hospital, Xi’an, Shaanxi, China.

**Keywords:** anti-tuberculosis drugs, multivariate linear regression analysis, serum concentrations, therapeutic drug monitoring

## Abstract

Therapeutic drug monitoring has been employed in anti-tuberculosis (TB) drugs to assess optimal dose for maximum therapeutic effects and minimal toxicity. But the determinants of serum concentration need further evidences.

In a retrospective case–control study, clinical and laboratory data were collected from 717 in-patients with TB at Xi’an Chest Hospital, China. Two hours serum concentrations of isoniazid, rifampicin, pyrazinamide as well as ethambutol were obtained and analyzed by liquid chromatography-tandem mass spectrometry.

The month 2 culture conversion group had lower concentration of isoniazid, pyrazinamide, and ethambutol than month 1 group. Statistical analysis showed that serum concentrations of isoniazid, rifampicin, pyrazinamide, and ethambutol revealed a positive relationship with dose (mg/kg) (*P* < .001, *P* < .001, *P* < .001, and *P* = .003, respectively). Furthermore, isoniazid concentration was related to smoking (*P* = .009) and prior TB (*P* = .011), while rifampicin and pyrazinamide concentrations were correlated to sex (*P* = .004 and 0.025, respectively). Ethambutol concentration was associated with creatinine clearance (Ccr, *P* = .002).

It is necessary to optimize drug doses using therapeutic drug monitoring while considering the following determinants: weight, smoking status, prior TB, sex, and Ccr. Furthermore, low 2 hours serum concentrations can be associated with longer culture conversion.

## 1 Introduction

Tuberculosis (TB) is the 9th leading cause of mortality worldwide as well as the 2nd leading cause of death resulting from a single infectious agent globally. In 2018, an estimated 10.0 million people were infected with TB, among whom 1.6 million died worldwide. China is among the countries with the highest TB burden, ranking 2nd in the world.^[1]^

In standard short-course chemotherapy, H isoniazid (INH), R rifampicin (RMP), Z pyrazinamide (PZA), and E ethambutol (EMB) are the commonly used first-line anti-TB drugs.^[2–5]^ However, treatment failure, relapse, drug toxicities, acquired drug resistance, and even multidrug-resistant TB have been reported among patients undergoing therapy.^[6]^

After decades of development, therapeutic drug monitoring has become an important clinical treatment for many diseases. Besides, several medical institutions have developed protocols for anti-TB drugs monitoring, which have benefited some TB patients.^[3]^ For some TB meningitis cases, overdose of anti-TB drugs may increase the burden of hepatic dysfunction, thrombocytopenia, and other adverse effects.^[7,8]^ Meanwhile, low serum concentration of anti-TB drugs would lead to poor outcomes, relapse, and drug-resistance.^[9–14]^ Previous studies showed that serum concentration of anti-TB drugs may be affected by factors such as age,^[15]^ gender,^[15–18]^ alcohol,^[19]^ consumption status, drug formulation,^[20]^ drug interactions,^[21]^ accompanying diseases such as HIV,^[22,23]^ hypoalbuminaemia,^[24]^ and dysfunctions of the liver and kidney.^[21]^ Low drug serum levels could be due to malabsorption,^[3,17]^ fast metabolism,^[9]^ and low dose per kg of body weight.^[15]^

The purpose of this study was to analyze the relationship between 2 hours serum levels of first-line anti-TB drugs (INH, RMP, PZA, and EMB) and the correlative factors, as well as to establish the preliminary relevance of the outcomes.

## 2 Methods

### 2.1 Chemicals and reagents

Mass-grade methanol, acetonitrile, formic acid, and ammonium formate were purchased from Fisher Scientific (Fair Lawn, NJ). HPLC-grade water was purchased from Watsons (Guangzhou, China). Paracetamol was purchased from Dr. Ehrenstorfer (Augsburg, Germany). INH was purchased from Yunpeng (Shanxi, China). RMP was purchased from Shuangding (Shenyang, China). PZA was purchased from Hongqi (Shenyang, China), whereas EMB was purchased from Minsheng (Hangzhou, China).

### 2.2 Clinical study

This study was implemented at Xi’an Chest Hospital and was approved by ethics committee of the hospital. This was a retrospective study without informed written consent. Among the patients admitted to the hospital from 1 August to 31 December 2018, eligible subjects were selected based on the following inclusion criteria for the study: hospitalized and received anti-TB therapy for at least 2 months; measured serum drug level after administration of the first-line anti-TB drugs daily for at least 1 week during hospitalization. To reduce the impact of outliers, patients were excluded if they were <15 years old, suspected of nonadherence, or had comorbid conditions such as liver or kidney dysfunction, HIV, and digestive tract diseases. Among the 717 enrolled patients, those that received treatment of H/R/Z/E, H/R/E, H/R/Z, and H/R were 495, 92, 89, and 41, respectively. According to the standard treatment regimen of WHO, most of TB patients received H/R/Z/E during intensive phase and H/R during consolidation phase. TB patients with negative sputum culture received H/R/Z during intensive phase. Relapse TB patients with positive sputum culture received H/R/E during consolidation phase. In addition, some combination treatments of patients were changed due to the adverse drug reactions.^[25]^ All enrolled patients were diagnosed with TB, via laboratory examination (such as sputum culture, Gene X-pert, etc), medical imaging, or clinical manifestation.

During hospitalization, the enrolled patients received the following daily oral doses: 300 mg INH; 450 mg RMP if they weighted <55 kg, 600 mg if they were heavier; 25 mg/kg PZA; and 15 mg/kg EMB. All these 4 drugs were single products.

Sputum culture data was collected at the time of admission, then after 1 month and 2 months in hospital. Culture conversion of month 1 group included patients who were initially positive but test negative after 1 month; month 2 group included patients who were positive in the initial and after 1 month, but tested negative in the 2nd months’ test or more. Other demographic, clinical, and laboratory characteristics were acquired from the patients’ record during admission period. The selection of covariates was based on the factors previously reported to influence the serum concentrations of first-line anti-TB drugs.^[15–21]^

### 2.3 Serum concentration analysis

Venous blood was collected 2 hours after oral drug ingestion (INH, RMP, PZA, and EMB). The blood was centrifuged at 1368 × g for 4 minutes in a low-speed centrifuge (ZONKIA, China). A total of 100 μL blood serum was subsided by 300 μL acetonitrile (containing 1 μg/mL paracetamol as internal standard). The mixture was vortex blended for 5 minutes in a thermo-shaker (Ausheng, China). After centrifugation at 14,000 × g for 10 minutes in a high-speed centrifuge (ZONKIA, China), the supernatant was diluted with 9 times volume of water.

The 5 μL aliquot was separated by Exion liquid chromatography (AB Science. Co., CA) with a Shim-pack XR-ODS column (100 × 2.0 mm, 2.2 μm, Shimadzu Co., Japan). The gradient elute of chromatographic separation was performed for 10 minutes with methanol, acetonitrile, water containing 0.1% formic acid, and 10 mmol/mL ammonium formate. Drug concentrations were quantified by an AB SCIEX API 3200 tandem mass spectrometer (AB Science. Co., CA) with ESI source in positive mode.

Concentrations of 0.2 to 10 μg/mL for INH and EMB, 1.5 to 30 μg/mL for RMP, and 4 to 70 μg/mL for PZA showed a repeatable linearity with correlation coefficient (*r*) greater than 0.9991. The 4 drugs were stable at the condition of 25 °C for 6 hours, 3 freeze/thaw between 24 hours, and −20 °C for 4 weeks. Their concentrations were all within 12.1% compared with the initial values whereas their relative standard deviation (SD) and accuracy of intraday were 4.9% to 10.3% and 89.3% to 109.8%, respectively. The relative SD and accuracy of inter- and intraday were 2.3% to 14.8% and 88.8% to 109.9%, respectively. Finally, the levels of unknown samples were calculated using the calibration curves and corrected by an internal standard. Quality control samples were prepared at high, medium, and low concentrations for each drug.

### 2.4 Reference range of INH, RMP, PZA, and EMB

Based on previous studies, most people reached a peak concentration after about 2 hours of drug ingestion.^[2,15]^ According to previously published data, the expected reference range for each of the drugs was: INH 3 to 6 μg/mL (300 mg daily); RMP 8 to 24 μg/mL (600 mg daily); PZA 20 to 60 μg/mL (weight-adjusted daily dose of 25 mg/kg); and EMB 2 to 6 μg/mL (weight-adjusted daily dose of 25 mg/kg).^[26]^

### 2.5 Statistical analysis

Normally distributed variables were described as mean ± SD, while nonnormally variables were described as median (Q1–Q3). For visual display of the data, some nonnormal variables were represented by mean ± SD. To identify the determinants of serum levels of anti-TB drugs, the reported factors were tested by univariate and multivariate linear regression analyses through a stepwise method. According to the normal distribution regulation of linear analysis, concentrations of the 4 drugs were log-transformed. Correlation analyses were expressed by Pearson correlation coefficient. For group comparison, the *t* test and one-way analysis of variance were applied for normally distributed variables, while the Mann–Whitney *U* test, Kruskal–Wallis test, and Spearman correlation were applied for nonnormally distributed variables. Categorical variables were analyzed using Chi-square test (2-sided). Statistical significance was considered at *P* < .05 and the data was interpreted using 95% confidence interval. SPSS 22.0 was used for data analysis (SPSS Inc., Chicago, IL).

## 3 Result

The demographic, clinical, and laboratory characteristics of the 717 enrolled patients are summarized in Table 1. For all the enrolled subjects, the median (range) age was 31 (16–90). We found that only 17.3% of the patients were tobacco smokers and 8.4% of them had a history of TB. To avoid poor absorption of fixed-dose combinations, all patients ingested single drug products. Low serum concentrations of INH, RMP, PZA, and EMB corresponding to 47.7% (342/717), 36.1% (222/615), 34.0% (147/432), and 45.5% (204/448), respectively, were observed. Among the 717 patients, the mycobacterium TB culture data of 35 patients was missed because of specimen collection problems; the sputum culture of 360 patients were consistently negative during the treatment; the sputum culture of 208 patients turned negative in 1 month (month 1 group); and the sputum culture of 114 patients turned negative in 2 months or more (month 2 group). To compared with month 1 group, the month 2 group included more male (*P* = .001), elder people (*P* = .016), smoker (*P* = .007), diabetes (*P* = .003), and patients with low serum albumin (*P* = .017). The concentration of INH (*P* < .001), PZA (*P* = .032), and EMB (*P* = .004) was lower in the cohort of month 2 compared to the cohort of month 1.

**Table 1:**
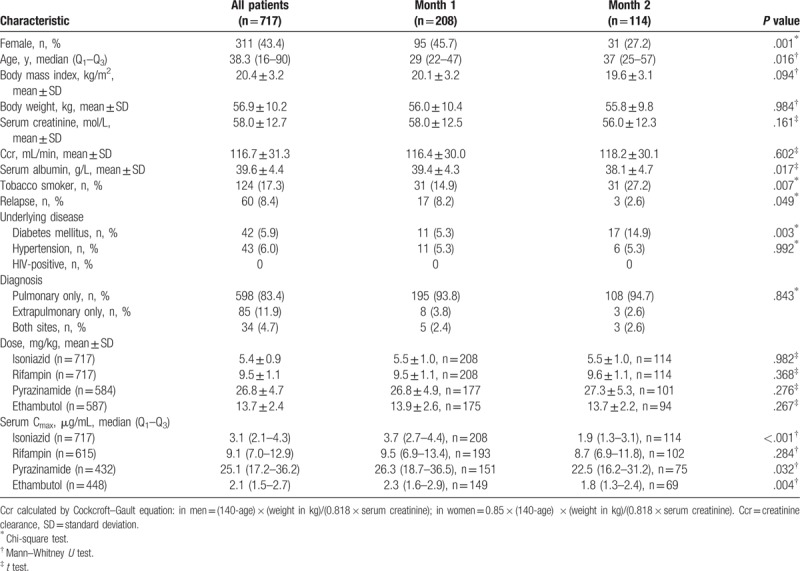
Demographic, clinical, and laboratory characteristics of 717 patients and different groups of culture conversion.

As shown in Table 2, group H/R/Z/E had a higher proportion of male (*P* = .022) and overall level of creatinine clearance (Ccr, *P* < .001) compared with other groups. By contrasted with group H/R/E and H/R, group H/R/Z/E and H/R/Z included more young patients, more smokers, but less relapses. Moreover, male patients revealed higher level of Ccr (*t* test, *P* < .001). Male included more smokers (Chi-square test, *P* < .001). In addition, relapses included more elder patients (*t* test, *P* = .003). The differences of patients with diabetes and hypertension in each group were related to the insufficient number of cases.

**Table 2:**
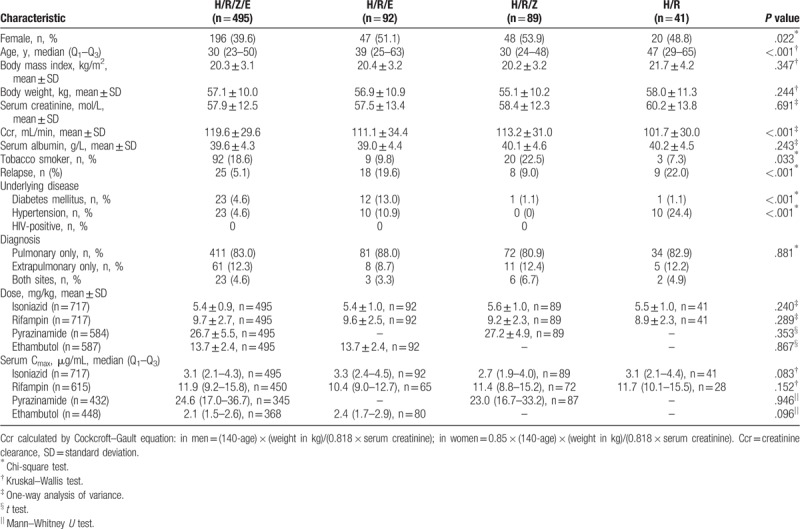
Demographic, clinical, and laboratory characteristics of patients for different dosing regimens.

### 3.1 Serum INH concentration

The concentration of 2 hours INH was significantly lower among smokers (n = 124, 3.0 ± 2.0 μg/mL) than nonsmokers (n = 593, 3.5 ± 1.9 μg/mL) according to Mann–Whitney *U* test (*P* < .001). Because of the majority proportion was male among smokers, the INH serum concentration of male smokers and male nonsmokers was compared. Male smokers (n = 122, 3.0 ± 2.0 μg/mL) had lower INH concentration than male nonsmokers (n = 284, 3.2 ± 1.7 μg/mL) according to Mann–Whitney *U* test (*P* = .035). Those with a history of TB (n = 60, 3.9 ± 1.9 μg/mL) showed higher INH concentration compared to those with initial TB (n = 657, 3.4 ± 1.9 μg/mL) by based on Mann–Whitney *U* test (*P* = .016). In addition, patients with a history of TB and those with initial TB displayed age (*β* = 0.124, *P* = .001) and Ccr (*β* = −0.071, *P* = .059) differences according to Spearman correlation analysis.

After analyses of all potential determinants shown in Table 1 by univariate linear regression, we found that the 2 hours serum INH concentration was associated with dose (mg/kg), sex, smoke status, and prior TB history (Table 3). Multivariate analyses for all variables found to be significant showed that: each INH dose enhancement of 1 mg/kg was associated with a 2 hours INH concentration increase of 0.249 g/mL (*P* < .001); and the INH concentration of smokers was lower than nonsmokers (*P* = .009). Patients with a history of TB showed higher INH concentration than those experiencing TB for first time (*P* = .011).

**Table 3:**
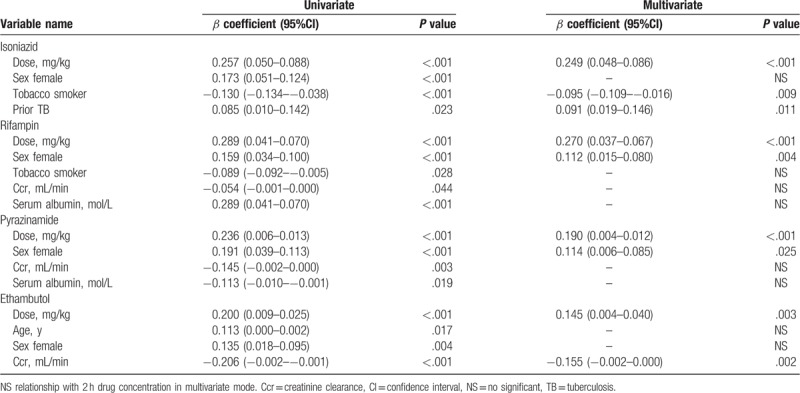
Determinants of 2 h serum isoniazid, rifampin, pyrazinamide and ethambutol concentration by univariate and multivariate analyses.

### 3.2 Serum RMP concentration

After screening all potential determinants, dose, sex, smoke status, Ccr, and serum albumin were founded to be associated with serum concentration of RMP based on univariate linear analysis (Table 3). Multivariate analysis showed that the 1 mg/kg increase of RMP for each dose was associated with a 2 hours RMP concentration increase of 0.270 μg/mL (*P* < .001), with females showing a higher RMP concentration than males (*P* = .004). Mann–Whitney *U* test of 2 hours RMP concentration revealed that there was a difference (*P* < .001) between females (n = 259, 11.1 ± 5.0 μg/mL) and males (n = 356, 9.6 ± 4.3 μg/mL).

### 3.3 Serum PZA concentration

Among all potential determinants, dose, sex, Ccr, and serum albumin were associated with serum concentration of PZA based on univariate linear analysis (Table 3). Multivariate analysis showed that 1 mg/kg increment of PZA for each dose was associated with a 2 hours PZA concentration increase of 0.190 μg/mL (*P* < .001), with females having a higher PZA concentration than males (*P* = .025). Mann–Whitney *U* test of 2 hours PZA concentration revealed difference (*P* < .001) between females (n = 190, 29.9 ± 12.6 μg/mL) and males (n = 242, 25.4 ± 11.7 μg/mL).

### 3.4 Serum EMB concentration

Based on univariate linear analysis, serum concentration of EMB was found to be associated with dose, age, sex, and Ccr (Table 3). Multivariate analysis showed that each 1 mg/kg increment of EMB dose was associated with an EMB concentration increase of 0.145 μg/mL (*P* = .003). Besides, each 1 mL/minute increase in Ccr caused an EMB concentration decline of 0.155 μg/mL (*P* = .002).

### 3.5 Relationship between serum concentrations and drug dosages

**Figure 1:**
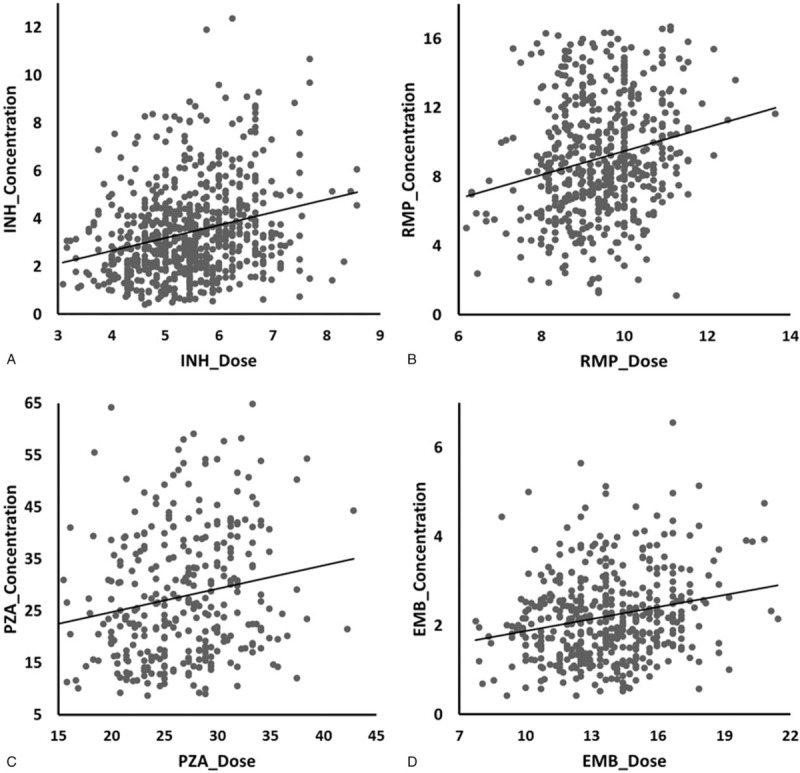
The scatterplots revealed that there was a positively correlation between the dose (mg/kg) and serum concentration of isoniazid (INH, A), rifampin (RMP, B), pyrazinamide (PZA, C), and ethambutol (EMB, D). The standard error of estimate, regression coefficient, and expected coefficient (95% confidence intervals) of the 4 drugs were: (A) 1.826, 0.266 (0.396–0.683); (B) 4.394, 0.300 (0.963–1.613); (C) 12.024, 0.227 (0.336–0.795); and (D) 0.987, 0.219 (0.053–0.128). The Pearson correlation coefficient of the 4 drugs was: (A) 0.257, *P* < .001; (B) 0.409, *P* < .001; (C) 0.236, *P* < .001; and (D) 0.200, *P* < .001. EMB = E ethambutol, INH = H isoniazid, PZA = Z pyrazinamide, RMP = R rifampicin.

Based on univariate and multivariate analyses, dosage was the most important determinant of serum concentration level. A scatterplot of the 4 anti-TB drugs with regard to the dose (mg/kg) and serum concentration is presented in Figure 1. The regression lines and Pearson correlation suggest that the dose (mg/kg) of each drug was positively correlated with its serum concentration, especially for RMP.

## 4 Discussion

The current study describes the distribution and possible determinants of serum concentration of INH, RMP, PZA, and EMB in 717 patients with TB in China. The results revealed that, month 2 conversion group attained lower concentration of INH, PZA, and EMB than month 1 group. It is consistent with previous prospective observational studies.^[27]^ Among these 4 drugs, there were at least 34.0% patients had 2 hours serum concentrations falling outside the normal range. Due to the influence of demographic characteristics such as gender and age, there were differences in bioinformatic analysis among 4 dosing regimens, but no differences in dosing dose and serum concentration. The scatter plot showed that patients with higher doses (mg/kg) would quite possibly present higher serum concentrations. Considering that all univariate and multivariate analyses revealed that serum concentrations were correlated with dose (mg/kg), this study suggests that the dosage of these 4 drugs should be adjusted in mg/kg instead of a stable dose, if their administration to the patients is not monitored. Previous studies also revealed that weight-adjust dose increased drug concentration.^[4,9]^ Furthermore, this study indicates that doses should be adjusted according to serum concentration results, because low serum concentration of anti-TB drugs is associated with poor outcomes.^[11,13,14,28,29]^

Many previous studies investigating the serum drugs concentrations required for effective therapy were only based on pharmacokinetic–pharmacodynamic data from human studies instead of real clinical outcomes.^[6,30]^ Besides, 2 hours serum drug measurement was not the actual peak serum concentration because of individual differences. Instead of normal area under the curve (AUC), limited sampling strategy should be paid more attention in the further studies, since it can predict with high accuracy and precision using the population pharmacokinetic model and C_max_ with limited sampling at 2, 4, and 8 hours after ingestion.^[31,32]^

According to this study, INH concentration was correlated with the dose (mg/kg), smoke status, and prior TB infection in both univariate and multivariate analyses. Due to the sex effect on smoke status, male smokers accounted for 98% of all smokers. Comparison with Mann–Whitney *U* test was used not only on smoker/nonsmoker, but also on male smoker/nonsmoker. The result showed that nonsmokers had higher INH concentration than smokers. The Mann–Whitney *U* test revealed that patients with prior TB had higher INH concentration than those with initial TB. Based on correlation analysis, patients with a history of TB were positively correlated with age, and negatively correlated with Ccr. Collectively, these factors may lead to a higher INH concentration in prior TB patients. Further prospective studies are needed to support the relationship between smoking, treatment history, and INH concentrations. Based on previous research, Asian people were generally rapid metabolizers of INH, thus further study should focus on the acetyl-INH/INH ratio.^[33]^ In other words, simultaneous monitored acetyl-INH/INH ratio may be a better way to approach the peak concentration of INH serum concentration.

According to previous studies, dose (mg/kg) and sex were associated with serum concentration of RMP and PZA.^[4]^ Our study supports this view point. Females showed higher concentrations of both RMP and PZA than males. On the base of multivariate analyses, serum concentration of EMB was related to the dose (mg/kg) and calculated Ccr. Since EMB is a renal excreted prototype drug, Ccr was inversely correlated with EMB concentration. In addition, most patients received doses of (13.7 ± 2.4 mg/kg) which were lower than the recommended dose of 25 mg/kg. Further, the 2 hours time period before collecting blood samples may be earlier for some patients than the recommended serum T_max_ of 2 to 3 hours.^[26]^

## 5 Conclusions

In summary, this study reveals that some patients have a low serum concentration of at least one drug, among the 4 anti-TB drugs. Low serum concentrations of INH, PZA, and EMB are associated with longer culture conversion. Several risk factors for low serum concentrations were determined to be dose (mg/kg), smoking status, relapse, gender, and Ccr. It is necessary to adjust dosages of these 4 first-line anti-TB drugs based on therapeutic drug monitoring for patients, especially for the patients with poor outcomes or with the problems of smoking, relapse, among others.

## Acknowledgments

The authors thank all of doctors, nurses, and laboratory staff of Xi’an Chest Hospital, who offered help on data collection and taking care of the patients.

## Author contributions

**Conceptualization:** Qian Lei, Hao Wang.

**Data curation:** Qian Lei, Hui Wang, Yuan Zhao, Jun Zhou.

**Formal analysis:** Qian Lei.

**Investigation:** Qian Lei.

**Methodology:** Qian Lei, Yuan Zhao, Jun Zhou.

**Project administration:** Qian Lei, Hao Wang, Liyun Dang, Changsheng Zhu, Xiaohui Lv.

**Resources:** Qian Lei, Liyun Dang.

**Software:** Qian Lei, Hao Wang, Hui Wang.

**Supervision:** Hao Wang, Liyun Dang, Changsheng Zhu, Xiaohui Lv.

**Writing – original draft:** Qian Lei, Hao Wang.

**Writing – review & editing:** Qian Lei, Hao Wang.
